# Male Decapacitation Factor SPINK3 Blocks Membrane Hyperpolarization and Calcium Entry in Mouse Sperm

**DOI:** 10.3389/fcell.2020.575126

**Published:** 2020-09-30

**Authors:** Lucia Zalazar, Cintia Stival, Anabella R. Nicolli, Gerardo A. De Blas, Dario Krapf, Andreina Cesari

**Affiliations:** ^1^Instituto de Investigaciones Biológicas (IIB), Consejo Nacional de Investigaciones Científicas y Técnicas (CONICET), Universidad Nacional de Mar del Plata, Mar del Plata, Argentina; ^2^Laboratory of Cell Signal Transduction Networks, Instituto de Biologia Molecular y Celular de Rosario (IBR), Consejo Nacional de Investigaciones Científicas y Técnicas (CONICET), Universidad Nacional de Rosario, Rosario, Argentina; ^3^Instituto de Histología y Embriología de Mendoza (IHEM), Consejo Nacional de Investigaciones Científicas y Técnicas (CONICET), Universidad Nacional de Cuyo, National Scientific and Technical Research Council, Mendoza, Argentina; ^4^Escuela Superior de Medicina, Universidad Nacional de Mar del Plata, Mar del Plata, Argentina

**Keywords:** decapacitation factor, sperm capacitation, membrane potential hyperpolarization, fertilization, SPINK3

## Abstract

Mammalian sperm acquire ability to fertilize through a process called capacitation, occurring after ejaculation and regulated by both female molecules and male decapacitation factors. Bicarbonate and calcium present in the female reproductive tract trigger capacitation in sperm, leading to acrosomal responsiveness and hyperactivated motility. Male decapacitating factors present in the semen avert premature capacitation, until detached from the sperm surface. However, their mechanism of action remains elusive. Here we describe for the first time the molecular basis for the decapacitating action of the seminal protein SPINK3 in mouse sperm. When present in the capacitating medium, SPINK3 inhibited Src kinase, a modulator of the potassium channel responsible for plasma membrane hyperpolarization. Lack of hyperpolarization affected calcium channels activity, impairing the acquisition of acrosomal responsiveness and blocking hyperactivation. Interestingly, SPINK3 acted only on non-capacitated sperm, as it did not bind to capacitated cells. Binding selectivity allows its decapacitating action only in non-capacitated sperm, without affecting capacitated cells.

## Introduction

After testicular ejaculation, mammalian sperm transit the female reproductive tract where they undergo a series of biochemical and physiological modifications in order to gain fertilization competence. These changes, known as capacitation (Austin, 1951; Chang, 1951), can also be promoted in culture medium containing Ca^2+^, HCO_3_^–^, energy sources, and a cholesterol acceptor such as BSA (Zalazar et al., 2012; Assis et al., 2013), mimicking an *in vivo* environment. Capacitation is a highly complex process in which influx of HCO_3_^–^ and Ca^2+^ plays a key role, acting on the atypical adenylyl cyclase sAC (aka ADCY10) (Hess et al., 2005; Stival et al., 2016). The activity of this cyclase increases intracellular cAMP concentration which promotes the direct activation of the Ser/Thr kinase Protein Kinase A (PKA), orchestrating different signaling cascades downstream (Aitken and Nixon, 2013). Its activation is involved in: membrane potential (*Em*) hyperpolarization and acquisition of acrosomal responsiveness, flagellar hyperactivation, intracellular alkalization, and phosphorylation of sperm proteins in Tyr residues, among other known effects (Puga Molina et al., 2018). Even though these processes differ in their kinetics, all of them involve phosphorylation cascades initiated by PKA. In this regard, we have recently shown that PKA activity is necessary for Src activation, which in turn leads to *E*m hyperpolarization and acrosomal responsiveness in mouse sperm (Stival et al., 2015).

In order for fertilization to succeed, capacitation must be highly time-controlled and synchronized with ovulation. Ejaculation prompts contact of spermatozoa with components of the seminal plasma. Some of these components have been proposed to bind to sperm and promote decapacitating activity (i.e., prevent capacitation), either negatively regulating or delaying capacitation. Detaching of these molecules allows capacitation to proceed. The protein SPINK3 (Serine Protease Inhibitor Kazal type 3) has been proposed as one of these decapacitation factors (Ou et al., 2012; Zalazar et al., 2012). SPINK3 is a 6 kDa protein with a well-characterized function as an inhibitor of trypsin-like serine proteases (Ohmuraya et al., 2012; Assis et al., 2013). However, among different functions ascribed to this protein, it has been found to reduce intracellular calcium increase ([Ca^2+^]i) that normally occurs upon capacitation (Coronel et al., 1992; Zalazar et al., 2012). The molecular mechanism underlying this effect is unknown. SPINK3 is secreted mainly by seminal vesicles into the seminal fluid where it adheres to the sperm surface (Chen et al., 1998) before entering the female duct (Ou et al., 2012). It has been hypothesized that decapacitating factors should detach from sperm for capacitation to proceed. Our results using the mouse model, showed that when sperm were challenged with SPINK3 and then exposed to capacitating conditions, capacitation failed to occur. Although phosphorylation of PKA substrates was not abolished, Src activation was impaired with the consequent lack of hyperpolarization. Accordingly, acrosomal responsiveness was not acquired. Calcium influx was also inhibited, and sperm did not acquire hyperactivation. Our data indicate that SPINK3 blocked both *Em* hyperpolarization and CatSper-dependent Ca^2+^ uptake, affecting the onset of capacitation. Supporting these results, sperm recovered from the uterus after mating showed binding of SPINK3 localized to the apical portion of the head and to the principal piece. Altogether, these results shed light on the molecular mechanism by which the seminal protein SPINK3 prevents premature capacitation in murine sperm.

## Results

### SPINK3 Inhibits the Acquisition of Acrosomal Responsiveness Associated With Capacitation

**FIGURE 1:**
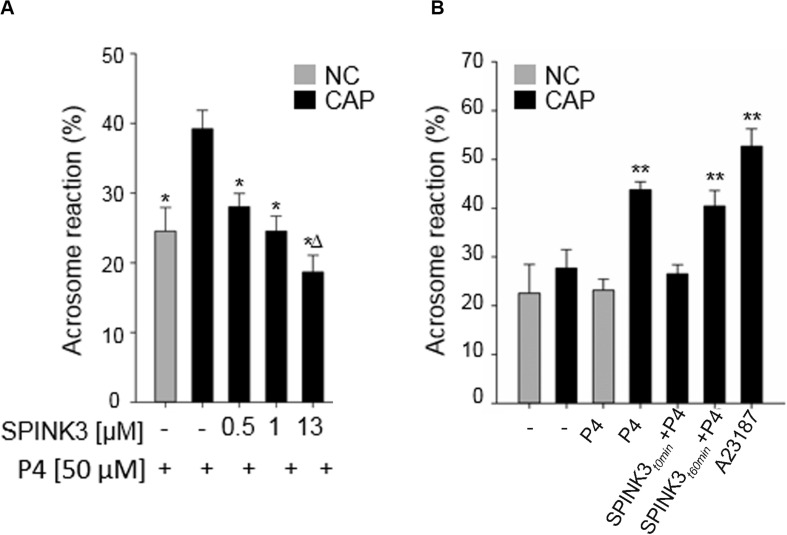
Effect of SPINK3 on acrosome reaction (AR). **(A)** Different SPINK3 concentrations were added at the beginning of the incubation time in either non-capacitating (NC, gray bars) or capacitating (CAP, black bars) medium (60 min at 37°C). AR was induced by 50 μM progesterone (“P4”) (data represent mean ± SE; *n* = 3). *p < 0.05 respect to control Progesterone-induced capacitated cells; Δ*p* < 0.05 compared to 1 μM SPINK3 treatment. **(B)** SPINK3 (13 μM) was added either at the beginning (SPINK3_t__0 min_ + P4) or at the end of capacitation (SPINK3_t__60 min_ + P4). Progesterone (50 μM) was always added after capacitation was completed (data represent mean ± S.E.; *n* = 8); **p < 0.05 with respect to cells treated with SPINK3 at the beginning of capacitation (SPINK3 + P4).

We have previously shown that the recombinant protein SPINK3 reduced acrosomal responsiveness in mouse sperm when added at the beginning of capacitation (Zalazar et al., 2012). Thus, in order to discard a blockade on the exocytotic mechanism *per se*, we added SPINK3 before or after capacitation. As shown in Figure 1A, when physiologically relevant SPINK3 concentrations (Dematteis et al., 2008; Samanta et al., 2018) were added to capacitating media from the beginning, induction of acrosome reaction (AR) with progesterone was impaired in a concentration dependent manner. Treatment with 13 μM SPINK3 showed complete AR blockade. On the other hand, when SPINK3 was added to capacitated sperm (i.e., after 60 min of incubation in capacitating medium), sperm underwent AR upon stimulation with progesterone (Figure 1B). These results suggest that SPINK3 inhibitory effect on AR is the consequence of sperm not achieving the capacitated state and therefore, not being able to acquire acrosome responsiveness.

### SPINK3 Disrupts Membrane Hyperpolarization Associated With Capacitation

**FIGURE 2:**
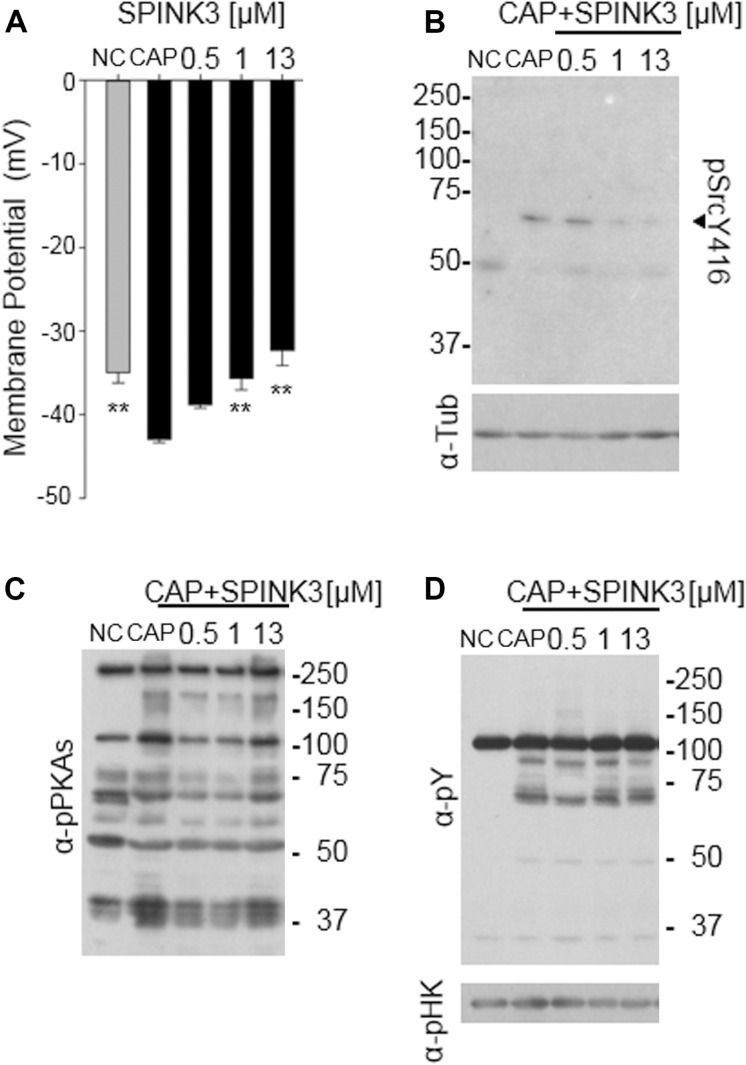
Effect of SPINK3 on sperm membrane potential (*E*m) and associated pathways. **(A)** *Em* measurements of DISC_3_(5)-loaded sperm, incubated for 60 min in non-capacitating (NC, gray bar) or capacitating (CAP, black bars) conditions containing the specified SPINK3 concentrations. Data represent mean ± S.E., *n* = 4, ** values significantly different (*p* < 0.01) compared to CAP; **(B,C)** after sperm incubations as detailed in **(A)**, western blot analyses were performed with anti pSrcY416 antibodies **(B)** (clone D49G4), further stripped and re-probed with anti-tubulin (α-Tub) antibodies. Alternatively, western blot analyses were performed with anti-pPKAs (clone 100G7E) **(C)**, further stripped and re-probed with anti-pTyr (α-pY) (clone 4G10) **(D)**. Western blot analyses are representative of experiments repeated at least three times. p-hexokinase (116 kDa, α-pHK) detected with anti-pY, is showed as loading control.

Since hyperpolarization of the plasma membrane potential (E*m*) has been proven to be necessary and sufficient for sperm to acquire acrosome responsiveness (De La Vega-Beltran et al., 2012), we hypothesized that SPINK3 was affecting this E*m* shift. The study of *Em* was performed on a fluorimeter using the probe DISC_3_(5) (Ritagliati et al., 2018a; Stival et al., 2015). The presence of SPINK3 in the capacitation medium significantly blocked sperm plasma membrane hyperpolarization in a concentration dependent manner (Figure 2A).

The tyrosine kinase Src activates during capacitation, and is necessary to achieve hyperpolarization of mouse sperm, modulating the K^+^ channel Slo3 (Stival et al., 2015). Thus, we analyzed whether Src activity was being affected by SPINK3. The activity of Src can be monitored by the phosphorylation status of its Tyr416, which is autophosphorylated upon activation. As shown in Figure 2B, phosphorylation of Tyr416 was not observed under the presence of SPINK3, arising as the molecular cause of the lack of hyperpolarization and acrosomal responsiveness.

Considering that PKA activity is upstream of Src activation (Stival et al., 2016), we hypothesized that SPINK3 effect on Src activation was due to decreased PKA activity. PKA can be easily monitored by western-blot assessment of phosphorylated PKA substrates (Krapf et al., 2010). As shown in Figure 2C, when SPINK3 was present in the medium, the overall pattern of phosphorylated PKA substrates remained comparable to that of the capacitation control, indicating that PKA activity was not substantially affected by the presence of SPINK3. In addition, no effect was observed on the phosphorylation status of tyrosine residues (Figure 2D), used for many years as a hallmark of sperm capacitation. These data portraits a situation where PKA is active, while Tyr416-Src phosphorylation is impaired by SPINK3 (1 μM and above) (Figure 2B), standing as a plausible cause for the absence of hyperpolarization.

Some evidence suggests that *Em* hyperpolarization is part of a complex mechanism of regulation (González-Martínez, 2003; Fraire-Zamora and González-Martínez, 2004; Del Carmen Neri-Vidaurri et al., 2006) in which interplay with calcium influx has been proposed (Orta et al., 2019). Therefore, the assessment of the effect of SPINK3 on intracellular calcium levels was pursued.

### SPINK3 Impairs Intracellular Calcium Increase During Capacitation

**FIGURE 3:**
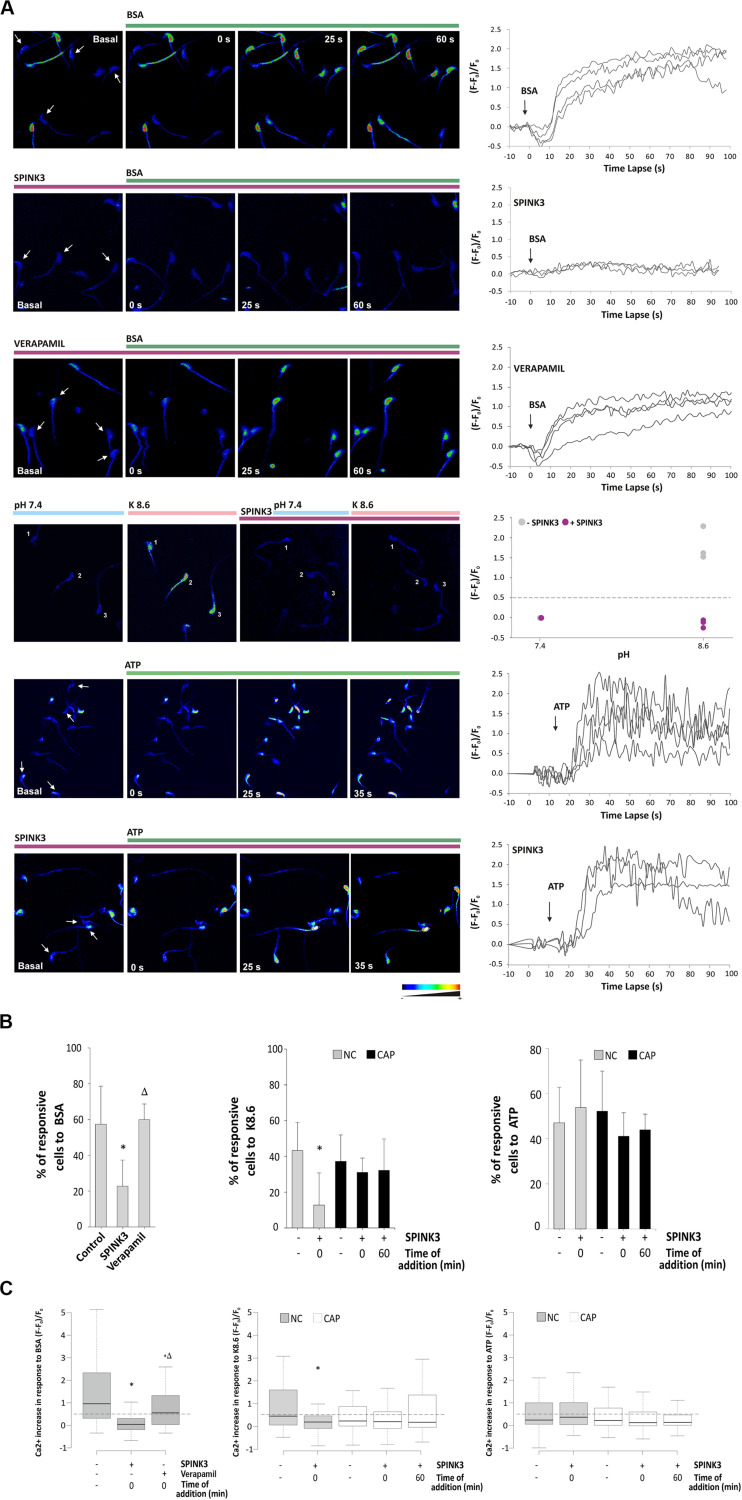
Effect of SPINK3 on [Ca^2+^]i during sperm capacitation. Non-capacitated sperm loaded with Fluo-3 AM were exposed to 13 μM SPINK3 or 100 μM verapamil for 15 min. After washing with fresh media, cells were smeared on laminin-coated slides and basal calcium signal (F_0_) was monitored before any stimulus was applied. Single-cell fluorescence (F) was monitored and recorded. **(A)** Representative pseudocolor images of calcium concentration in the head of control cells. Different stimuli (BSA, K8.6 medium or ATP) were added to test an intracellular calcium increase (green bar). Graph on right panels show representative images for calcium fluctuations, obtained as the fluorescence change (F - F_0_)/F_0_. **(B)** Percentages of sperm that increased at least 1.5-fold the calcium intensity compared to the basal signal after stimulus. Capacitated (CAP) or non-capacitated (NC) sperm were treated as indicated (left panel, BSA: *n* = 12, 822 cells, *p < 0.05 with respect to control, Δ*p* < 0.05 with respect to SPINK3; middle panel K8.6: *n* = 3, 430 cells, *p < 0.05 with respect to control; right panel: ATP: *n* = 3, 410 cells, *p < 0.05 with respect to control). **(C)** Fluorescence intensity after 60 s of stimulus expressed as (F - F_0_)/F_0_, where F is the fluorescence intensity at time t and F_0_ is the mean basal fluorescence. The dotted line indicates the threshold lay out from which a significant increase in calcium uptake in the sperm head (positive response) was considered. Outliers were excluded from the graphical representation. Capacitated or non-capacitated sperm were treated as indicated (left panel, BSA: *n* = 12, 822 cells, *p < 0.05 with respect to control, Δ*p* < 0.05 with respect to SPINK3; middle panel K8.6: *n* = 3, 430 cells, *p < 0.05 with respect to control; right panel: ATP: *n* = 3, 410 cells, *p < 0.05 with respect to control).

Considering that SPINK3 affected signaling events conducive to capacitation associated hyperpolarization, we investigated whether intracellular calcium increase was affected by SPINK3. By using a single-cell imaging approach, we studied SPINK3 effect on intracellular calcium exerted by BSA, which is known to induce CatSper-mediated Ca^2+^ entry (Xia and Ren, 2009). CatSper is a sperm-specific calcium channel with a key role in the acquisition of fertilization competence. Cells were loaded with the calcium sensitive probe Fluo3-AM, and equilibrated in medium supplemented with NaHCO_3_. Upon addition of BSA, there was an increase of intracellular calcium signal of living cells. Even though the influx of Ca^2+^ was noticeable 20 s after BSA addition, the highest signal intensities were reached after 60 s post-stimuli (Figure 3A, upper panel), as previously reported (Ren and Xia, 2010). About half of the living sperm analyzed in control conditions exhibited a positive response to stimulus (Figure 3B), as expected from the high heterogeneity of sperm populations (Movie S1). When sperm were pre-incubated with SPINK3, the increase in calcium signal upon BSA induction was significantly inhibited (Figure 3A second panel, Figures 3B,C and Supplementary Movie S2) compared to untreated cells. Basal [Ca^2+^]i was not affected by pre-incubation of sperm with SPINK3 considering the ratio between treated/non-treated cells (1.06 ± 0.4). As a second approach, the effect of SPINK3 over CatSper Ca^2+^ entry was addressed by means of depolarization coupled to alkalization (K8.6 solution treatment), in order to elicit a CatSper-dependent [Ca^2+^]i increase in mouse sperm (Xia et al., 2007). K8.6 did not promote intracellular Ca^2+^ increase in non-capacitated (“NC”) cells incubated with SPINK3, as evidenced by the reduction in the percentage of responsive cells and in the amount of the calcium intensity (Figure 3A fourth panel, Figures 3B,C middle panel). However, SPINK3 failed to inhibit this response in capacitated sperm (“CAP”) when challenged with K8.6 (Figures 3B,C middle panel). These results indicated that SPINK3 was affecting calcium entry through CatSper channel only when present from the beginning of capacitation. To rule out the possible role of the purinergic P2X receptor channels (Navarro et al., 2011), non-capacitated sperm where exposed to 2.5 mM ATP either in the presence or absence of SPINK3. Both conditions showed normal calcium response (Figure 3A last two panels and Figures 3B,C right panels). In addition, spermatozoa showed normal response to BSA when incubated in the presence of Verapamil, an L-type calcium channel blocker (i.e., does not affect CatSper) (Figure 3A third panel and Figures 3B,C left panels). These data indicate that SPINK3 blocked calcium influx presumably through CatSper channels.

**FIGURE 4:**
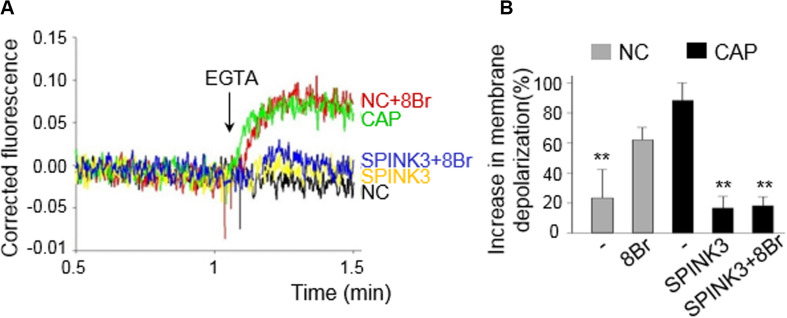
Effect of SPINK3 on Sodium-dependent depolarization induced by EGTA. CatSper opening was indirectly assessed by membrane potential (*Em*) recordings, and following the magnitude of the EGTA-induced depolarization. Capacitated (CAP) or non-capacitated (NC) sperm were loaded with 1 μM DISC_3_(5) during *Em* recordings. **(A)** Representative traces are shown for all conditions assayed: CAP (green), NC (black), CAP + 13 μM SPINK3 (yellow); NC + 1 mM 8Br-cAMP (red); CAP + 13 μM SPINK3 + 1 mM 8Br-cAMP (blue). **(B)** The increase in membrane depolarization is expressed as (F - F_0_)/F_0_ × 100, where F_0_ is the mean value before EGTA addition, and F represents the DISC_3_(5) signal after addition of EGTA. **p < 0.01 with respect to CAP.

In order to analyze the effect of SPINK3 on CatSper opening, cells were capacitated in either the presence or absence of SPINK3 and then loaded with DISC_3_(5) (Kirichok et al., 2006; Carlson et al., 2009; López-González et al., 2014; Ritagliati et al., 2018b; Stival et al., 2018; Torres-Flores et al., 2011). After fluorescence stabilization, calcium was chelated with 3.5 mM EGTA (Bers et al., 1994; Ritagliati et al., 2018b) allowing sodium to permeate through CatSper. The increase in fluorescence after addition of EGTA indicates the magnitude of depolarization caused by sodium influx, and correlates to the extent of CatSper opening. As shown in Figure 4, treatment with 13 μM SPINK3 effectively blocked EGTA-induced depolarization. Recent evidence suggests that CatSper could be stimulated by PKA in mouse sperm (Stival et al., 2018; Orta et al., 2019). Thus, cells treated with SPINK3 were also incubated in the presence of the cAMP permeable-analog 8Br-cAMP to directly activate PKA, and consequently CatSper. However, EGTA-induced depolarization could not be rescued by directly activating PKA with 8Br-cAMP, further substantiating that SPINK3 effect is not due to PKA downregulation.

### SPINK3 Binds to Non-capacitated Cells

**FIGURE 5:**
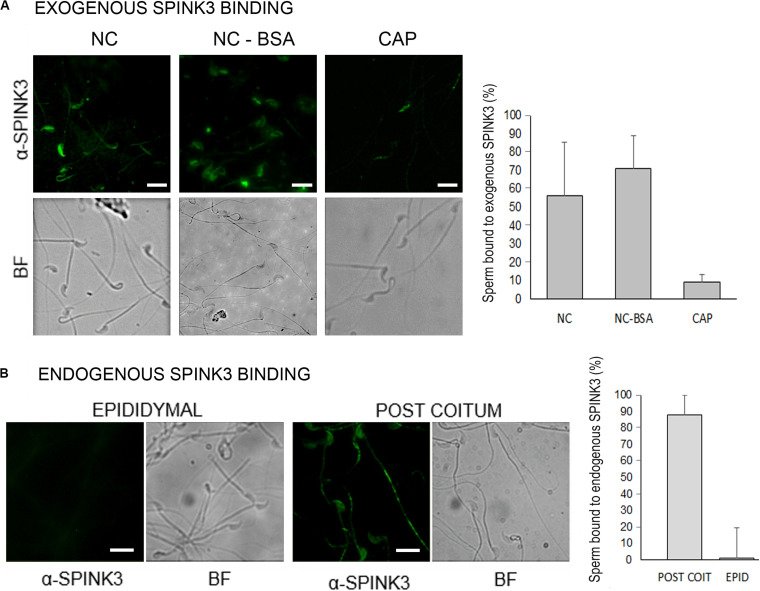
Distribution of SPINK3 on capacitated and non-capacitated sperm. **(A)** Caudal sperm were incubated in non-capacitating (NC) or capacitating (CAP) conditions and then treated with recombinant SPINK3 (13 μM, “exogenous SPINK3”) for 15 min, washed and fixed. As a control of BSA blocking effect, a NC + BSA condition was included. SPINK3 was immunodetected as detailed in Methods. **(B)** Sperm recovered from the uterus of synchronized females immediately after coitus or from the male epididymis (negative control of cells that were not in contact with seminal vesicle secretions) were fixed. Then, native SPINK3 (“endogenous SPINK3”) was immunodetected. For **(A,B)** left panel shows representative pseudocolor images and bright fields (BF), and right panel shows percentage of positive immunoreactive cells. Scale bar = 10 μM.

SPINK3 negatively regulates hyperpolarization presumed to be driven by SLO3 potassium channels (Santi et al., 2010) and also, either directly or indirectly, CatSper channels. Considering that both SLO3 and CatSper locate exclusively to the principal piece of the flagellum (Navarro et al., 2007; Chung et al., 2014; Hwang et al., 2019), we studied whether recombinant SPINK3 binds to this region. Sperm from cauda epididymis incubated under non-capacitating conditions in the presence of recombinant SPINK3, showed SPINK3 binding to the principal piece and head. However, after *in vitro* capacitation, sperm failed to bind to exogenously added SPINK3 (Figure 5A). Lack of SPINK3 binding to capacitated sperm was not due to BSA masking because both capacitated and non-capacitated sperm were incubated with BSA and further washed before SPINK3 addition. As a control, non-capacitated cells exposed to BSA still bound SPINK3 (Figure 5A). This *ex vivo* experiment was replicated by an *in vivo* approach, to assess the binding of endogenous SPINK3 to naturally deposited sperm in females, after matting. Immediately after coitus, sperm were recovered from the female uterus and analyzed for the presence of SPINK3. Under these conditions, a strong signal was observed in the sperm principal piece. Worth noticing, control sperm derived from the cauda epididymis that were never in contact with endogenous SPINK3, showed no immunofluorescence signal (Figure 5B).

### SPINK3 Inhibits Acquisition of Hyperactivation

**FIGURE 6:**
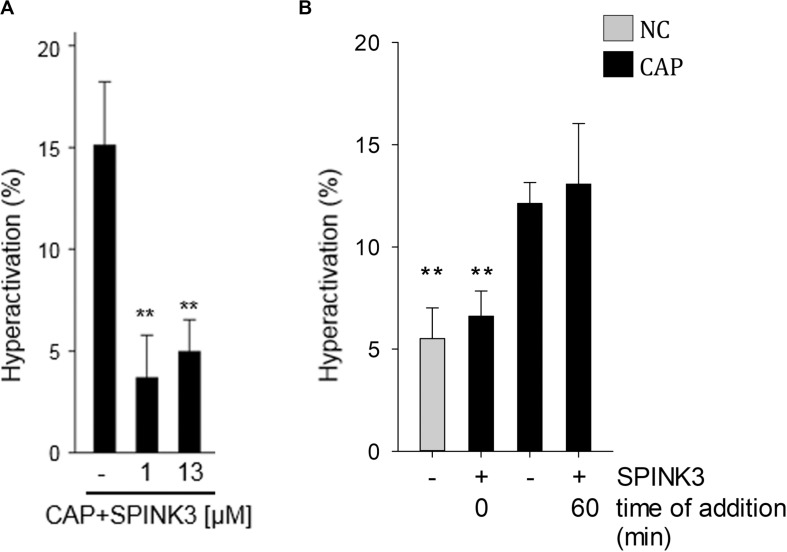
Effect of SPINK3 on hyperactivation. **(A)** Mouse sperm were incubated in the absence or presence of different SPINK3 concentrations in medium that supports capacitation (CAP). Hyperactivation was analyzed using a Hamilton Thorne IVOS CASA system (data represents mean ± SE, *n* = 3; **p < 0.01 with respect to CAP control). **(B)** Sperm were incubated under non-capacitating (NC) or capacitating (CAP) medium for 75 min, and 1 μM SPINK3 was added either since the beginning (0 min) or at the end (60 min) of the capacitation period (data represents mean ± S.E.M., *n* = 3; **p < 0.01 with respect to CAP control).

Hyperactivation is a functional change in the sperm movement pattern, associated to capacitation and described as a relatively progressive motion with high-amplitude flagellar bending (Yanagimachi, 1970). Calcium is essential for hyperactivation of mouse sperm (Carlson et al., 2003). Therefore, the effect of SPINK3 on acquisition of hyperactivation was evaluated. While no statistically significant differences were observed in total motility upon supplementation of capacitating media with SPINK3 (Zalazar et al., 2012), hyperactivation was impaired by 1 μM SPINK3 and above (Figure 6A). No effect of SPINK3 was observed on hyperactivation when SPINK3 was added to already capacitated sperm (Figure 6B). This result further substantiates the role of SPINK3 as a protein that prevent acquisition of fertilizing competence by impairing hyperpolarization and dysregulation of calcium entry.

## Discussion

Sperm leave the male body through ejaculation, as part of a complex suspension where seminal proteins are found. Among these proteins, decapacitation factors are important constituents of semen, but still poorly understood (Araki et al., 2015). One of these factors is the protein SPINK3, present in the seminal vesicles at a concentration of approximately 11 μM (Coronel et al., 1992). SPINK3 is known as a serine protease inhibitor Kazal type 3. Upon secretion by the seminal vesicle, SPINK3 binds to ejaculated sperm (Chen et al., 1998; Zalazar et al., 2012) until it apparently detaches in the uterus during ovulation (Ou et al., 2012). Similarly, other decapacitation factors with protease inhibitory activity have been described with a similar behavior (Lin et al., 2008; Li et al., 2010). Increasing evidence ascribes to SPINK3 functions other than a protease inhibitor, e.g., growth factor for regeneration of pancreatic acinar cells and cell proliferation during early embryogenesis (Ohmuraya et al., 2005, 2006). Although SPINK3 lacks calcium-binding domains that could act as Ca^2+^ chelator (Marchler-Bauer and Bryant, 2004; de Castro et al., 2006; Mazumder et al., 2014), it has been described as a Calcium Transport Inhibitory protein (CalTrIn) because it could reduce intracellular calcium content in sperm (Coronel et al., 1992; Zalazar et al., 2012). Our manuscript shows that SPINK3 is capable of impairing sperm membrane hyperpolarization and calcium influx through CatSper when present during capacitation at physiologically relevant concentrations, substantiating the molecular basis of its action as a decapacitating factor. However, no protease target for SPINK3 inhibitory action has been identified.

The mechanisms that regulate hyperpolarization of the sperm plasma membrane during capacitation are poorly understood. Using knock-out mouse models it was observed that sperm lacking the sperm-specific K^+^ channel SLO3 do not undergo hyperpolarization during capacitation. We have previously shown that activation of the tyrosine kinase Src is necessary for hyperpolarization of the mouse sperm plasma membrane (Stival et al., 2015), as it modulates SLO3 function. Thus, the fact that SPINK3 prevented activation of Src, substantiates the hypothesis that SPINK3 prevents hyperpolarization through lack of SLO3 opening. Hyperpolarization was shown to be required for exocytosis of the sperm acrosome (De La Vega-Beltran et al., 2012). In this regard, the inhibition of acrosome responsiveness by SPINK3 could be explained by the lack of hyperpolarization. Accordingly, sperm lacking SLO3 do not undergo acrosome reaction when stimulated (Chávez et al., 2013).

Two types of Ca^2+^ channels have been reported to be present in mouse sperm flagellum; the sperm-specific voltage-dependent CatSper channel (at the principal piece) and P2X receptor channels (purinergic Ca^2+^ channels, at the midpiece). However, only CatSper was shown to be active during capacitation (reviewed by Lishko and Mannowetz, 2018). The fact that Ca^2+^ cytoplasmic increase was abrogated by SPINK3, suggests that normal function of CatSper is being impaired by SPINK3. It has been proposed that the elevation of Ca^2+^ necessary to hyperactivate the sperm is primarily driven by an interplay of CatSper and SLO3 channels (Navarro et al., 2007; Zeng et al., 2011, 2013). However, the functional relationship between the two channels has not yet been completely demonstrated. Activation of CatSper by SLO3 appears to be indirect and may involve a voltage-dependent change in intracellular pH (Kirichok et al., 2006; Chávez et al., 2013). Our data indicate that when SPINK3 is present since the beginning of capacitation, Ca^2+^ influx through CatSper is impaired. This conclusion is supported by the following evidence: (a) BSA fails to elicit intracellular Ca^2+^ increase when SPINK3 is present; (b) SPINK3 does not abolish Ca^2+^ increase when sperm are prompted with ATP, suggesting that P2X channels are not involved, (c) Ca^2+^ increase caused by alkalization/depolarization triggered by K8.6 solution is affected by the presence of SPINK3, and (d) SPINK3 decreases the depolarization induced by monovalent ions influx when EGTA is added to the extracellular media. Whether SPINK3 affects calcium entry through an indirect effect on SLO3 appears as a plausible explanation, although a direct effect cannot be discarded, based on the effect seen by SPINK3 on K8.6 prompted sperm. Further supporting a possible direct effect, SPINK3 effect on CatSper could not be rescued by directly activating PKA with 8Br-cAMP, substantiating that SPINK3 effect is not due to PKA downregulation. It should be noted that cAMP analogs could also act directly on CatSper, through extracellular binding (Wang et al., 2020).

Calcium influx mediated by CatSper is required for essential sperm functions, including hyperactivation (Lishko and Mannowetz, 2018). In this regard, SPINK3 also affected the acquisition of the hyperactivated motility. However, SPINK3 affected neither progesterone-induced acrosome reaction nor hyperactivation when added after capacitation has been completed. This might reflect the lack of SPINK3 binding to capacitated sperm, as supported by our immunofluorescence data. Single-cell analysis showed that [Ca^2+^]i increase observed when sperm were prompted with different stimuli was not homogeneous throughout the population; half of the sperm were responsive to BSA stimulus and among them, half of these cells were not affected by the presence of SPINK3. The existence of sperm sub-populations was already described (Buffone et al., 2004; Sousa et al., 2011; Cisneros-Mejorado et al., 2014), and was therefore not surprising. In this regard, SPINK3 could not bind equally to all spermatozoa, as seen by immunofluorescence assays probably due to different degrees of sperm epididymal maturation. In addition to different maturational status, a selective binding mechanism of SPINK3 to non-capacitated cells over capacitated sperm would evidence its role as decapacitating factor. Therefore, SPINK3 could allow triggering of capacitation at proper timing while not affecting cells that have already initiated capacitation.

In conclusion, this work presents molecular insights into the decapacitating effect exerted by the seminal plasma protein SPINK3 on mouse sperm. Its presence in the capacitation medium prevented both acquisition of hyperactivation and acrosomal responsiveness and inhibits the acquisition of sperm hyperpolarization associated to sperm capacitation. This effect involves a deficient Ca^2+^ uptake through CatSper, which altogether stands out as the main mechanism for its decapacitation effect. Thus, sperm encounter SPINK3 during ejaculation, preventing off-site capacitation. Whether the environment of the female tract contributes to SPINK3 detachment, allowing capacitation to proceed still needs to be addressed.

## Materials and Methods

### Reagents

Secondary antibodies were from Cell Signaling Technologies, Fluo-3 AM and Pluronic acid F127 from Invitrogen, United States. Laminin was acquired from BD. All other chemicals and reagents were analytical grade and obtained either from Merck or Sigma-Aldrich.

### Animals

BalbC and C57-BL mice (*Mus musculus*) were maintained at 22°C with a photoperiod of 12 h light: 12 h darkness, food and water *ad libitum*. Sexually mature (2–3 months old) male mice were euthanized by cervical dislocation or by CO_2_, inhalation, and sperm from caudal epididymides were removed. All procedures were in agreement with the local Ethics Committee of the National University of Mar del Plata (RD 225/16), as well as the Animal Care and Use Committee of the National University of Rosario (RD 7298/532), following the National Institutes of Health Guide for the Care and Use of Laboratory Animals^1^.

### Heterologous Expression of Recombinant SPINK3

The cDNA encoding the mature SPINK3 from *Mus musculus* (NCBI ID: NM 009258.5) was cloned into the pET-24b (+) (Novagen, United States) expression vector. Overexpression of SPINK3 was performed in *Escherichia coli* Rosetta cells (Novagen, United States) and the recombinant protein was purified to apparent homogeneity by a HiTrap IMAC HP (GE Healthcare Life Sciences, United States) affinity chromatography as was described in a previous work Assis et al. (2013). Purified recombinant protein was dialyzed against phosphate buffer saline (PBS, 10 mM phosphate buffer, 137 mM NaCl and 2.7 mM KCl pH 7.4).

### Sperm Preparation

*Cauda* epididymides were immersed in HM medium (Modified Krebs Ringer medium without bicarbonate: 25 mM Hepes, 109 mM NaCl, 14.77 mM KCl, 1.19 mM MgSO_4_, 5.6 mM glucose, 21.18 mM sodium lactate, 1.19 mM KH_2_PO_4_, 1.2 mM sodium pyruvate and 1.7 mM CaCl_2_; pH 7.4) placed in culture dishes on a warm plate at 37°C. The tissues were minced with scissors to allow the sperm dispersion into the media. After 10 min, tissue debris were removed, and caudal non-capacitated sperm were washed with fresh HM medium by mild centrifugation. For capacitation, 25 mM NaHCO_3_ and 3 mg/ml BSA (HMB) was added, and sperm incubated in an atmosphere of 5% CO_2_ at 37°C for 90 min, at 7.5 × 10^6^ cells/ml, as indicated in the corresponding assays. In cases where capacitation was performed in the presence of either 0.5, 1, or 13 μM SPINK3, the peptide was added to the sperm suspension from the beginning of the incubation time (t_0_) or after capacitation (t_60_), as indicated in each experiment.

### Time Lapse Single-Cell Imaging

Non-capacitated sperm (10 × 10^6^ cells/ml) were loaded with 10 μM Fluo-3 AM (Invitrogen United States, Molecular Probes F1242) and 0.02% (v/v) pluronic acid (Invitrogen, United States) as a surfactant at 37°C for 30 min in HM medium. After incubation, sperm were washed by centrifugation (3 × 700 × *g*, 5 min) to remove unincorporated dye. To evaluate the effect of different compounds, sperm were pre-incubated during 15 min in non-capacitating medium at 37°C in the presence or absence of 13 μM SPINK3. Unless indicated, the assay was performed with non-capacitated spermatozoa. As a control, cells were treated with 100 μM Verapamil (a L-type calcium channel blocker). Cells from an aliquot of each treatment were adhered onto a chamber with a slide previously covered with laminin (100 μg/ml, BD Biosciences) to ensure the head sperm adherence to the glass. HM medium supplemented with 25 mM NaHCO_3_ was added to the chamber and basal fluorescence was monitored under confocal microscope (excitation 488 nm; emission 526 nm, Nikon C1SiR) at 400 x magnification. A stimulus of BSA (3 mg/ml final concentration) or extracellular ATP (2.5 mM, Pharmacia, United States) was applied, and changes in calcium signal were video recorded throughout the process at the times indicated. At specified times, 10 μM A23187 was added to identify responsive cells. Fluorescence signal intensity was quantified at the head of sperm with beating flagella by using ImageJ 1.43 free software (National Institutes of Health, Bethesda, Maryland, United States)^2^. Data were normalized using the equation (F - F_0_)/F_0_, where F is the fluorescence intensity at time t and F_0_ is the mean fluorescence taken during the control period (initial 10 s) before the addition of either BSA or ATP. Then, total series of (F - F_0_)/F_0_ plotted vs. time as well as the percentage of positive response to stimulus. Threshold to consider a significant increase in calcium uptake was as previously reported (Xia and Ren, 2009). For alkalization assay, cells were incubated under the appropriate conditions and seeded onto laminin coated slides in a perfusion chamber. The chamber was filled with HM medium supplemented with 25 mM NaHCO_3_ (pH 7.4) and basal fluorescence was monitored. The increase in calcium after a change in the pH by addition of K8.6 solution (135 mM KCl, 5 mM NaCl, 2 mM CaCl_2_, 1 mM MgCl_2_, 10 mM glucose, 10 mM lactic acid, 1 mM sodium pyruvate, 30 mM HEPES, pH 8.6 (Wennemuth et al., 2000) was evaluated. Data were normalized using the equation (F - F_0_)/F_0_, where F is the fluorescence intensity after addition of K8.6 medium and F_0_ is the basal fluorescence. Cells that increased at least 1.5-fold the calcium intensity after K8.6 medium were quantified.

### Immunolocalization of SPINK3

For detection of recombinant SPINK3 binding to capacitated or non-capacitated sperm, caudal sperm were obtained in HM. After washing, cells were incubated either in HM (NC), HM plus 3% BSA (NC-BSA) or HMB (CAP) for 60 min. Then, sperm were washed (700 x g, 10 min) with PBS and incubated with or without 13 μM recombinant SPINK3 for 15 min, washed, fixed with 4% (v/v) formaldehyde solution and placed onto glass slides. Sperm were blocked in PBS plus 3% (w/v) BSA for 1 h, and then incubated overnight with anti-SPINK1 antibody (1:50, Sigma-Aldrich, HPA027498, this antibody reacts against SPINK3 (Zalazar et al., 2012) in blocking solution. Thereafter, slides were gently washed and incubated for 2 h with both anti-rabbit IgG Alexa Fluor 555 conjugated (1:1,000, Thermo Fisher Scientific). Slides were washed, mounted with Glicerol:PBS (9:1) and observed under 480/525 nm filter on a microscope: Nikon Eclipse T2000. The specificity of the antibody was assessed in samples without SPINK3, and secondary antibody control was performed by incubating without the respective primary antibodies. For the detection of endogenous SPINK3, sperm were recovered from the uterus of synchronized females immediately after coitus or from the male epididymis (negative control of cells that were not in contact with seminal vesicle secretions), washed (700 × *g*, 10 min) with PBS and fixed with 4% (v/v) formaldehyde solution. Immunolocalization assay was performed as was previously described by using anti-SPINK1 antibody (1:50, Sigma-Aldrich, HPA027498) and anti- rabbit IgG Alexa Fluor 488 (1:1,000, Thermo Fisher Scientific, A11070).

### Evaluation of Acrosome Reaction (AR)

Caudal sperm were capacitated in HMB medium for 60 min, with or without SPINK3. For induced AR, sperm were incubated with 50 μM progesterone (Zalazar et al., 2012) or 10 μM A23187 (Sigma-Aldrich, United States) for 15 min after 60 min capacitation. To evaluate the effect of SPINK3 over AR once capacitation was completed, cells were capacitated at 37°C for 1 h without SPINK3 and thereafter SPINK3 was added for 15 min before progesterone induction of AR. Cells were fixed with 4% (v/v) formaldehyde solution in PBS and washed with 100 mM ammonium acetate (pH 9). The acrosomal status was evaluated according to the *Coomassie Brilliant Blue* (CBB) G-250 staining technique (Bendahmane et al., 2002). Briefly, sperm were placed and smeared onto glass slides and stained with a 0.22% (w/v) CBB G-250 solution prepared in 50% (v/v) methanol and 10% (v/v) acetic acid. The slides were washed with distilled water, dried, smeared on glycerol: PBS (9:1) and observed under a light microscope at 400 x magnification by two independent observers. A blue stain over the sperm head dorsal and/or ventral edge was visualized in spermatozoa with intact acrosome, whereas no stain was observed in spermatozoa with reacted acrosome. Spermatozoa that lost their acrosomes were quantified as a percentage over 200–400 cells per replicate.

### SDS-PAGE and Immunoblotting

Samples were prepared as described previously (Ritagliati et al., 2018b). Briefly, sperm were collected after treatments by centrifugation, washed in 1 ml of TBS, resuspended in Laemmli sample buffer without β-mercaptoethanol, and boiled for 3 min. After centrifugation, 5% (v/v) β-mercaptoethanol was added to the supernatants and boiled again for 5 min. Protein extracts equivalent to 1–2 × 10^6^ spermatozoa per lane were subjected to SDS-PAGE and electro-transferred to PVDF membranes (Bio-Rad) at 250 mA for 60 min on ice. Membranes were blocked with 5% (w/v) fat free milk in TBS containing 0.1% (v/v) Tween-20 (T-TBS). For anti-pY and anti-pPKA immunodetections, membranes were blocked with 3% (w/v) BSA (Sigma) in T-TBS. Antibodies were diluted in T-TBS as follows: 1/10,000 for anti-pY (clone 4G10 Millipore), 1/5,000 for anti-pPKA (clone 100G7E Cell Signaling Technology), 1/10,000 for anti-tubulin (clone E7, Hybridoma bank), anti 1/2,000 pSrcY416 (Cell Signaling Technology clone D49G4). Secondary antibodies were diluted 1/10,000 in T-TBS and developed using an enhanced chemiluminescence detection kit (ECL plus, Amersham, GE Healthcare) according to the manufacturer’s instructions. When necessary, PVDF membranes were stripped at 60°C for 15 min in 2% (w/v) SDS, 0.74% (v/v) β-mercaptoethanol, 62.5 mM Tris, pH 6.5, and washed 6 × 5 min in T-TBS. In all experiments, molecular masses were expressed in kDa. Western blots showed in the manuscript are representative of at least three replicates.

### Membrane Potential Assay in Cell Populations

After sperm treatment, cells were collected by centrifugation (700 × g, 5 min) and concentration was adjusted to 2.7 × 10^6^ sperm/ml. Then, sperm were loaded with 1 μM of the membrane potential-sensitive dye DISC_3_(5) (Molecular Probes) for 5 min. No mitochondrial un-couplers were used because their contribution to the resting potential has been determined to be insignificant (Chávez et al., 2013). The sperm were transferred to a gently stirred cuvette at 37°C, and the fluorescence was monitored with a Varian Cary Eclipse fluorescence spectrophotometer at 620/670 nm excitation/emission wavelengths. Recordings were initiated when steady-state fluorescence was reached and calibration was performed at the end of each measure by adding 1 μM valinomycin and sequential additions of KCl as previously described (Ritagliati et al., 2018a). Sperm *Em* was obtained from the initial fluorescence (measured as arbitrary fluorescence units) by linearly interpolating it in the theoretical *Em* values for the calibration curve against arbitrary fluorescence units of each trace. This internal calibration for each determination compensates for variables that influence the absolute fluorescence values.

### Sodium Dependent Depolarization Induced by EGTA as a Measure of Calcium Channels Activity

For experiments where EGTA-induced depolarization was assessed, extracellular Ca^2+^ was chelated using EGTA allowing sodium ions influx through CatSper to occur (Kirichok et al., 2006). The magnitude of the depolarization caused by Na^+^ (present in the culture media) influx relates to the extent of the channel opening. Cells were incubated during capacitation as required, and then loaded with 1 μM DISC_3_(5). Fluorescence was recorded as detailed above. Calcium was chelated with 3.5 mM EGTA to a free calcium value of 138 nM (MaxChelator) (Patton et al., 2004). The fluorescence change was presented as (F - F_0_)/(F_0_), where F represents fluorescence intensity after EGTA addition, F_0_ is the mean of 1 min of acquisition before addition of EGTA (Stival et al., 2018).

### Sperm Motility Analysis

Sperm suspensions were loaded so as to form a 40 μM depth chamber slide and placed on a Hamilton Thorne IVOS CASA analyzer (Hamilton Thorne Research, Beverly, MA), at 37°C. Parameters used were as follows: 30 frames acquired, frame rate of 60 Hz, minimum cell size of 4 pixels, low average path velocity cutoff of 10 mm/s, static head size of 0.43–2.43, static head intensity of 0.53–1.52, and static head elongation lower than 100. At least 10 microscopy fields corresponding to a minimum of 200 sperm were analyzed in each experiment. Hyperactivated sperm were classified as such when presenting; Curvilinear Velocity (VCL) ≥271 μM/s, Linearity (LIN) <50% and Amplitude of Lateral Head displacement (ALH) >7 μM.

### Statistical Analysis

Data from all experiments were analyzed by GLMM (generalized linear mixed effect model) to determine statistical significance between treatments and control (Zuur et al., 2009). Data associated to cell percentages were analyzed through models with binomial distribution, whereas fluorescence intensities were analyzed by models with Gaussian error distribution. In both cases, normality of residuals was assessed by plotting theoretical quantiles vs. standardized residuals (Q–Q plots). Homogeneity of variance was evaluated by plotting residuals vs. fitted values. A *post-hoc* analysis was conducted with the “lsmeans” package (Lenth, 2016). All analyses were performed using R software version 3.3.3^3^, with the “lme4” package for binomial models and the “nlme” package for Gaussian models (Pinheiro et al., 2017). For all analyses, statistically significant differences were determined at *p* <0.05. Graph bars indicate mean ± SE.

## Data Availability Statement

All datasets generated for this study are included in the article/Supplementary Material.

## Ethics Statement

The animal study was reviewed and approved by the Ethics Committee of the National University of Mar del Plata (RD 225/16), as well as the Animal Care and Use Committee of the National University of Rosario (RD 7298/532).

## Author Contributions

AC and DK contributed to the conceptualization of the study, supervision, and project administration. LZ, CS, GD, and AN contributed to the design and development of the methodology. AC, DK, LZ, and CS contributed to the writing – reviewing and editing of the manuscript. All the co-authors were involved in the formal analysis, validation, and visualization of the results.

## Conflict of Interest

The authors declare that the research was conducted in the absence of any commercial or financial relationships that could be construed as a potential conflict of interest.

## Footnotes

1 http://grants.nih.gov/grants/olaw/Guide-for-the-Care-and-Use-of-Laboratory-Animals.pdf

2 http://imagej.nih.gov/ij/

3 https://CRAN.R-project.org/package=nlme

## Supplementary Material

MOVIE S1 Time course of the Ca^2+^ fluorescence changes with BSA stimulus. Non-capacitated sperm were loaded with Fluo-3 AM and smeared onto laminin coated slides. Basal calcium signal was monitored for 10 s after addition of BSA stimulus had been applied.

MOVIE S2 Time course of the Ca^2+^ fluorescence changes with BSA stimulus in the presence of SPINK3. Non-capacitated sperm were loaded with Fluo-3 AM and incubated with 13 μM SPINK3 during 15 min at 37°C before being smeared onto laminin coated slides. Basal calcium signal was monitored for 10 s after addition of BSA stimulus had been applied.
